# A Reticulated Rash With Rapid Recovery

**DOI:** 10.7759/cureus.81788

**Published:** 2025-04-06

**Authors:** Dana Simon, Michelle Gallagher

**Affiliations:** 1 College of Osteopathic Medicine, Michigan State University College of Osteopathic Medicine, East Lansing, USA; 2 Pediatric Dermatology, Michigan State University College of Osteopathic Medicine, East Lansing, USA

**Keywords:** acanthosis nigricans, carp, confluent and reticulated papillomatosis, hyperpigmented reticular rash, reticulated rash

## Abstract

Confluent and Reticulated Papillomatosis (CARP) is a rare and benign dermatosis, however, it can cause immense cosmetic insecurities in affected patients. It is most common on the trunk and neck, more common in female patients than male patients, and there appears to be a correlation with overweight and obese body mass index levels. Many of the patients presenting with CARP are adolescents who are especially vulnerable to resultant social isolation and embarrassment due to the cosmetic insecurities they face from CARP. For this reason, prompt diagnosis and treatment of the condition is critical. There are many conditions that can mimic CARP, with acanthosis nigricans being the most common, however, CARP has a much more direct and timely treatment option. We present a case of a 13-year-old female patient with a complaint of a three-year history of stable discoloration to the chest and neck who was able to achieve clearance in just eight weeks with the appropriate treatment.

## Introduction

Confluent and Reticulated Papillomatosis (CARP) is a rare and benign dermatosis, typically presenting as a tan reticulated hyperpigmentation. Prevalence rates are not well established. It most commonly affects the trunk and submammary creases, and the prognosis is good with adequate treatment. The most common treatment for CARP is minocycline. CARP presents at the time of adolescence, and is seemingly more common in patients who are female, black, overweight, obese, or have preexisting acanthosis nigricans [1]. The etiology and pathogenesis of CARP is not fully understood, but the following theories have been proposed: abnormal keratinization [2], microbial involvement [3], insulin resistance [4], and sweat gland dysfunction [4]. There are many conditions that can mimic CARP, and differential diagnoses consist of acanthosis nigricans, dermatopathia pigmentosa reticularis, macular amyloidosis, tinea versicolor, and terra firma-forme dermatosis. This case report highlights a 13-year-old female patient presenting to the pediatric dermatologist with a three-year history of discoloration to the chest and neck diagnosed as CARP who ultimately achieved full clearance of the pigmentation, following an eight-week course of antibiotics.

## Case presentation

**Figure 1:**
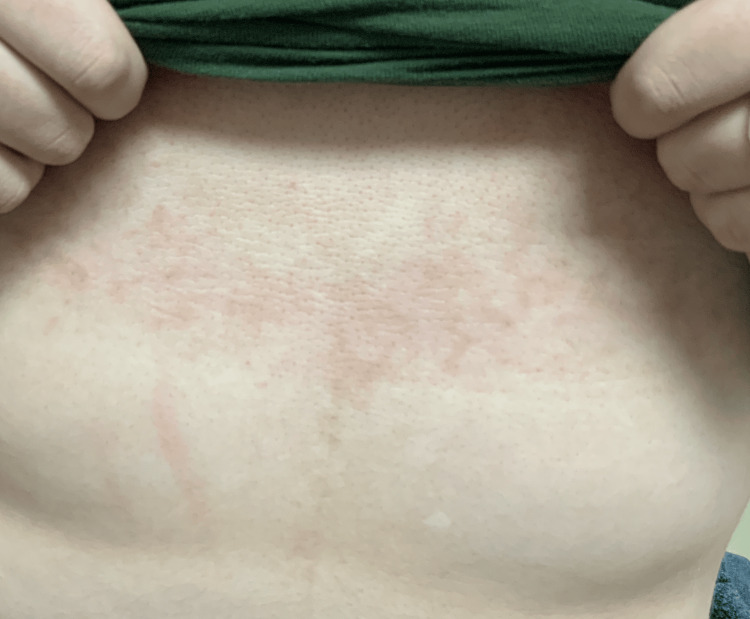
Hyperpigmented papules and plaques in a reticular pattern in subxiphoid region.

**Figure 2:**
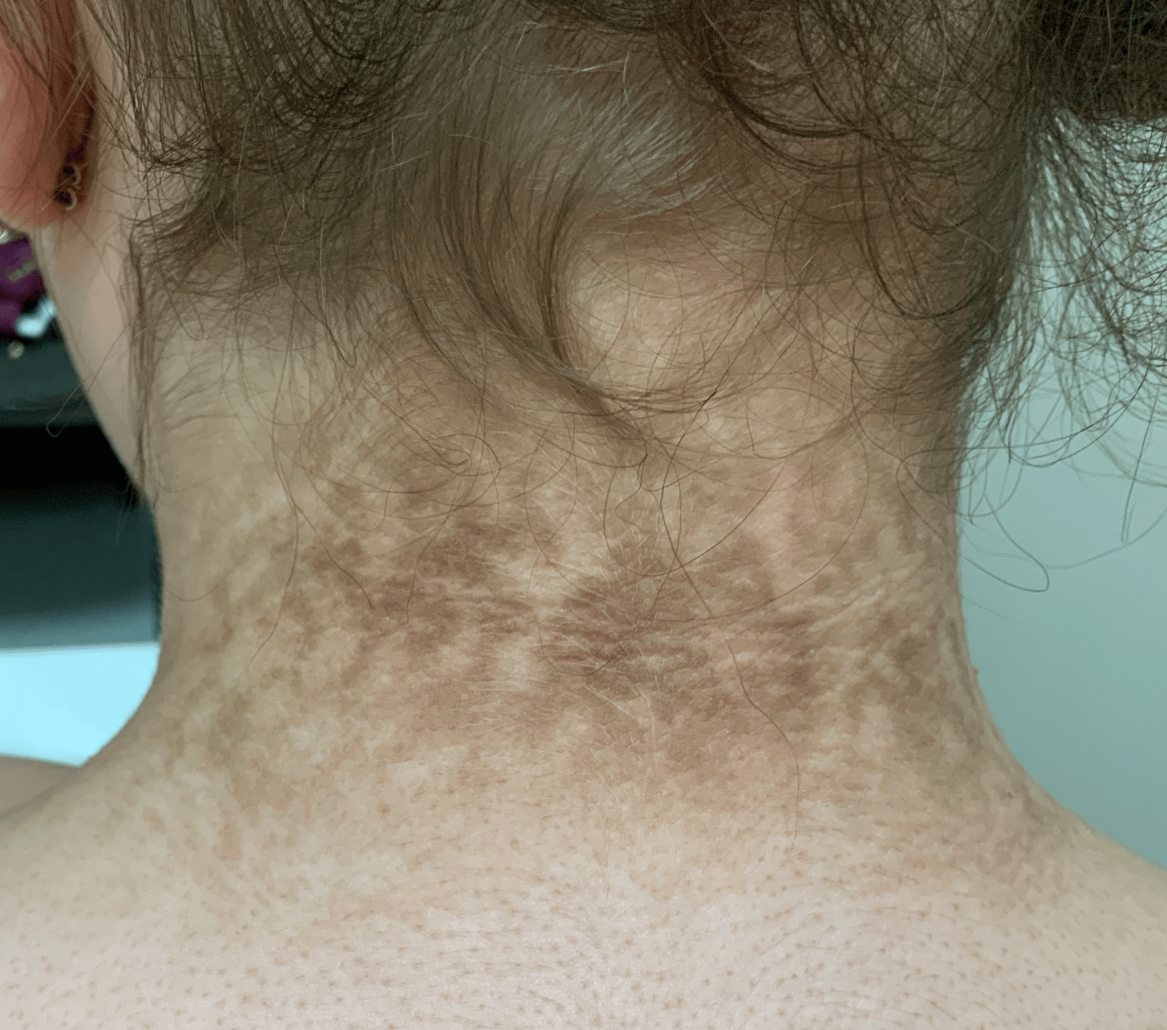
Hyperpigmented papules and plaques in a reticular pattern on posterior neck.

A 13-year-old female, with no significant medical history, presented with a three year history of discoloration on the chest, left breast, and left anterior neck. There had been no changes to the rash over 3 years, and she denied associated symptoms. She had not previously attempted any treatments. The patient was 82.5 kg and 152 cm, placing her in the obese category for body mass index, and the patient did not know how long she had been considered obese. The physical exam revealed hyperpigmented papules and plaques in a reticular pattern, distributed on the subxiphoid (Figure 1), left rib cage, posterior neck (Figure 2), right axillary vault, and left axillary vault. There was no hair or nail involvement. She was comfortable and in no distress throughout the examination.

**Figure 3:**
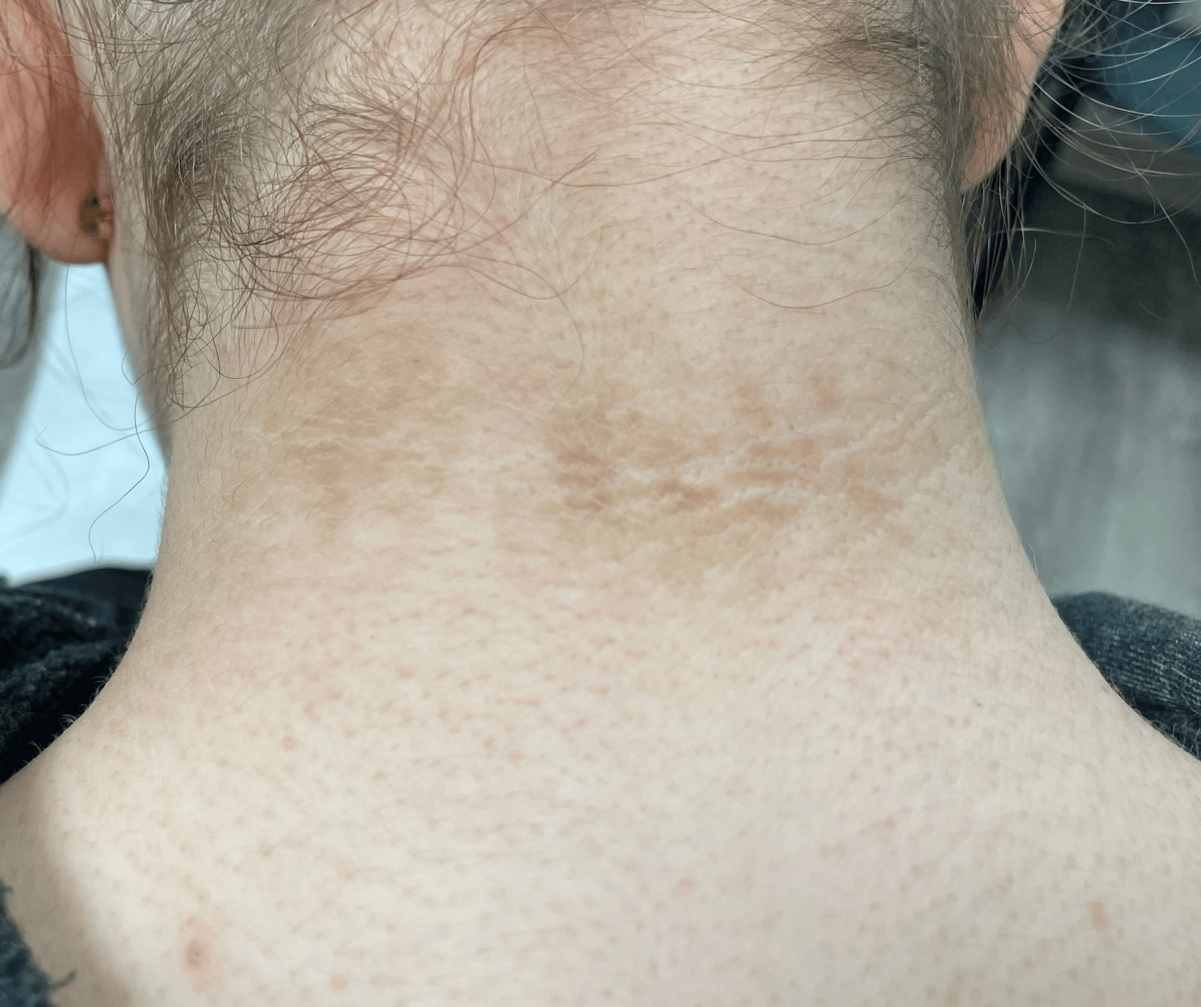
Rapid clearance and almost full resolution of hyperpigmented reticular rash at her four-week follow-up appointment.

Based on the appearance of the discoloration, the presence of the rash in the intermammary region, and the reticulated pattern, CARP was the suspected diagnosis, and the patient was started on oral minocycline 100 mg twice a day. Due to complaints of headaches and blurry vision, she was switched to oral azithromycin 250mg, three times weekly, which effectively cleared the dermatosis in eight weeks (Figure 3). The prompt clearance of the pigmentation following antibiotic treatment provided reassurance of our CARP diagnosis.

## Discussion

CARP is most commonly misdiagnosed as acanthosis nigricans, and differentiating the two is critical in order to facilitate prompt management for patients who may be dealing with insecurities. The urgency is especially important due to the target population of this skin disorder being adolescents, as they are increasingly vulnerable to cosmetic insecurities. Of note, CARP consists of reddish brown hyperpigmentation due to increased melanosomes within the hyperkeratotic stratum corneum, whereas acanthosis nigricans appears as more tan or brown color which looks like velvety plaques. In addition, CARP typically starts in the intermammary region and spreads outwards, whereas acanthosis nigricans is more common in the axilla, and seems to have a more intense pigmentation. Although successful clearance following antibiotics is a common and non-invasive way to confirm the diagnosis of CARP, histological evaluation can be used which would show milder epidermal changes in CARP compared to acanthosis nigricans [5]. In our patient, the hyperpigmentation was noted to be reddish, especially in the intermammary region, which helped solidify the diagnosis of CARP versus acanthosis nigricans.

A wide variety of treatment options have been successfully used to clear CARP, including topical keratolytics, topical antifungals, and most commonly, antibiotics [6]. Oral antibiotics have been proven to be more effective for CARP than topicals. Minocycline is typically the treatment of choice, but it can have an extensive side effect profile, as exhibited in our patient [6]. While the exact bacterial organisms contributing to CARP have not been established, minocycline shows efficacy in the treatment of CARP, which is why it was the first drug of choice for our patient. Potential side effects include vestibular dysfunction, drug-induced lupus, drug-induced hypersensitivity syndrome, hyperpigmentation, and dyspigmentation. In the event of an adverse side effect, azithromycin, doxycycline, or cefdinir are some effective alternative options. While these drugs do have a more favorable side effect profile compared to minocycline, they have not proven equal efficacy to minocycline in the treatment of CARP, which is why they are not the first-line choice. With that said, relapse of the condition has been reported upon discontinuation of treatment, and periodic exacerbations and remissions should be expected [7].

It has been reported that the disease state of CARP lasts longer in adults compared to adolescents, but histological changes are more prominent in adolescents compared to adults [8]. Histopathology findings of CARP consist of hyperkeratosis, acanthosis, and papillomatosis [9]. The dermis may also show perivascular lymphocytic infiltrate [10]. While histological evaluation is helpful in a diagnosis of CARP, it is typically diagnosed clinically, as was the case in our patient.

## Conclusions

This patient’s presentation highlights a rare, but important diagnosis of CARP. It is important for physicians to provide prompt treatment in the most effective way for our patients, and the early recognition of CARP can decrease the number of total doctor visits for the patient and improve the quality of their care. CARP has many mimickers, but it is easy to treat, so it should be included in a list of differential diagnoses to allow a hyperpigmented reticulated rash the chance for a rapid recovery. Our patient was young and insecure about her rash, and a rapid diagnosis and treatment was extremely important to her.
